# Screening and treatment for *Staphylococcus aureus* in patients undergoing hemodialysis: a systematic review and meta-analysis

**DOI:** 10.1186/1471-2369-15-202

**Published:** 2014-12-18

**Authors:** Cibele Grothe, Mônica Taminato, Angélica Belasco, Ricardo Sesso, Dulce Barbosa

**Affiliations:** Paulista School of Nursing, Universidade Federal de São Paulo (Federal University of São Paulo - EPE/UNIFESP), R. Napoleão de Barros 754, São Paulo, 04024-002 Brazil; Division of Nephrology, Paulista School of Medicine, Universidade Federal de São Paulo (Federal University of São Paulo - EPM/UNIFESP), R. Botucatu 740, São Paulo, 04023-900 Brazil

**Keywords:** *Staphylococcus aureus*, Colonization, Infection, Hemodialysis, Treatment

## Abstract

**Background:**

This study was performed to evaluate the effectiveness of surveillance for screening and treatment of patients with chronic kidney disease undergoing hemodialysis and colonized by *Staphylococcus aureus*.

**Methods:**

A systematic review and meta-analysis were performed. The literature search involved the following databases: the Cochrane Controlled Trials Register, Embase, LILACS, CINAHL, SciELO, and PubMed/Medline. The descriptors were “*Staphylococcus aureus*”, “MRSA”, “MSSA”, “treatment”, “decolonization”, “nasal carrier”, “colonization”, “chronic kidney disease”, “dialysis”, and “haemodialysis” or “hemodialysis”. Five randomized controlled trials that exhibited agreement among reviewers as shown by a kappa value of >0.80 were included in the study; methodological quality was evaluated using the STROBE statement. Patients who received various treatments (various treatments group) or topical mupirocin (mupirocin group) were compared with those who received either no treatment or placebo (control group). The outcomes were skin infection at the central venous catheter insertion site and bacteremia.

**Results:**

In total, 2374 patients were included in the analysis, 626 (26.4%) of whom were nasal carriers of *S. aureus*. The probability of *S. aureus* infection at the catheter site for hemodialysis was 87% lower in the mupirocin group than in the control group (odds ratio [OR], 0.13; 95% confidence interval [CI], 0.05–0.34; p < 0.001). The risk of bacteremia was 82% lower in the mupirocin group than in the control group (OR, 0.18; 95% CI, 0.08–0.42; p < 0.001). No statistically significant difference in bacteremia was observed between the various treatments group (excluding mupirocin) and the control group (OR, 0.77; 95% CI, 0.51–1.15; p = 0.20).

**Conclusions:**

Twenty-six percent of patients undergoing hemodialysis were nasal carriers of *S. aureus*. Of all treatments evaluated, topical mupirocin was the most effective therapy for the reduction of *S. aureus* catheter site infection and bacteremia in patients undergoing chronic hemodialysis.

**Electronic supplementary material:**

The online version of this article (doi:10.1186/1471-2369-15-202) contains supplementary material, which is available to authorized users.

## Background

*Staphylococcus aureus* is the most frequently isolated pathogen in hospitals worldwide. Approximately 20% of healthy people are chronic carriers of *S. aureus*, 30% are intermittent carriers, and 50% are not susceptible to carriage for unknown reasons [1].

*Staphylococcus aureus* infection has become endemic in health care institutions worldwide. Up to 70% of infections occur in such institutions. The reported half-life of colonization may reach 40 months in individuals who do not receive treatment [2]. An estimated 2 million individuals are carriers of *S. aureus* based on prevalence data in the Netherlands, while an estimated 53 million are carriers in the United States [3].

In one study, the cloning results of *S. aureus* isolated from blood were identical to those of *S. aureus* isolated from nasal specimens in 82.2% of patients undergoing hemodialysis (HD). These findings suggest that in these patients, the organisms isolated from the blood originated from the nasal flora [1].

There are few reports on infection and colonization of *S. aureus* because few countries conduct epidemiological surveillance of this microorganism. Information on colonization can only be obtained when there is an active search for carriers because colonization is asymptomatic. After patients with chronic kidney disease (CKD) undergoing conservative treatment are colonized with *S. aureus* during hospitalization, these patients may persist as carriers for a prolonged period of time, even after hospital discharge. They may subsequently reintroduce the bacteria into the hospital during another hospital admission [4].

The greatest risk of transmission occurs when the patient is not identified as a carrier. In a retrospective analysis that evaluated information from hospitalizations during an 8-year period, a German hospital evaluated the risk of acquiring *S. aureus* by patients who shared the same hospital room with other colonized, but unidentified, patients. The study found a 13% incidence of infection by the same strain of *S. aureus* among colonized patients [5]. Another study evaluated the prevalence of *S. aureus* on admission and found that 49% of carrier patients would not have been identified without screening on admission [1].

Infectious complications caused by *S. aureus* are common, and an increased frequency is being observed. Such complications include bacteremia, endocarditis, osteomyelitis, and metastatic abscesses [6]. The overall mortality rate associated with *S. aureus* bacteremia ranges from 11.9% to 46.5% [7].

Research performed in our institution demonstrated that application of the topical antibiotic mupirocin at the venous catheter insertion site significantly reduced the risk of colonization and bloodstream infection with *S. aureus*; moreover, the same result was observed in another study conducted in Australia [8, 9]. A systematic literature review and meta-analysis published in 2011 including 8 randomized controlled trials involving 3396 participants demonstrated that nasal decolonization of patients with *S. aureus* using mupirocin led to a significant reduction in infections caused by this microorganism [10]. However, the emergence of strains resistant to multiple drugs, including mupirocin, should be considered, such as that demonstrated in a study from Spain [11].

Collection of surveillance cultures has been advocated as a way to control the dissemination of multidrug-resistant pathogens. Early detection of patients colonized with multidrug-resistant microorganisms may permit the effective establishment of measures to control cross-transmission [12].

Eradication of the carrier state of *S. aureus* includes prevention of both infection and transmission. Several eradication strategies have been evaluated, but these studies differed significantly in their design.

To fill these knowledge gaps, we conducted a systematic review to evaluate the effectiveness of surveillance for screening of *S. aureus* in patients with CKD and determine the most effective intervention with which to eradicate the transmission of this bacterium among patients with CKD undergoing HD.

## Methods

This systematic review and meta-analysis follows the steps proposed by the Cochrane Collaboration [13] and is in accordance with PRISMA guidelines statement for systematic review reporting [14]. The inclusion criterion was *S. aureus* colonization in patients with CKD undergoing HD as the primary outcome. The exclusion criteria were nonrandomized studies, letters, editorials, and case reports; studies involving patients <18 years of age; evaluation of *S. aureus* infection treatment outcomes without evaluation of the effect of nasal colonization; and no specification of the therapy administered to the treatment group. The interventions compared in this meta-analysis were surveillance for screening of nasal carriers of *S. aureus*, prophylactic treatment/decolonization to control cross-transmission, and *S. aureus* infection (bacteremia and skin infection at the catheter insertion site) between treated and untreated patients undergoing HD.

The following characteristics of each study were extracted: study design; total numbers of patients receiving various treatments (various treatments group), mupirocin (mupirocin group), and placebo or control (control group) with corresponding rates of eradication; colonization by *S. aureus*; duration of surveillance; number of patients with skin infection at the catheter insertion site; and episodes of bacteremia.

### Study identification strategy

Relevant studies published from January 1989 to January 2014 were identified through a search of the following electronic databases: the Cochrane Library (including the Cochrane Controlled Trials Register), Embase, LILACS, SciELO, CINAHL, and Medline/PubMed. The principal descriptors used in the search were “*Staphylococcus aureus*”, “MRSA”, “MSSA”, “treatment”, “decolonization”, “nasal carrier”, “colonization”,”chronic kidney disease”, “dialysis”, and “haemodialysis” or “hemodialysis”.

### Study selection

The studies were read by two independent reviewers (C.G. and M.T.) to ascertain whether they fulfilled the inclusion criteria. The reviewers were not blinded. Each reviewer evaluated the titles and abstracts of all identified studies and obtained complete photocopies of all relevant articles. In cases of doubt or disagreement, a third reviewer (D.A.B.) was solicited to issue an opinion regarding whether the study should or should not be included.

### Evaluation of methodological quality

Methodological quality was defined as confidence that the study design and reporting were free of bias. Two independent reviewers used the recommendations of the Cochrane framework and the STROBE statement (STrengthening the Reporting of OBservational studies in Epidemiology). Based on the STROBE recommendations [13], studies included in the meta-analysis were divided into three categories: (A) >80% compliance with the STROBE criteria, (B) 50% to 80% compliance with the STROBE criteria, and (C) <50% compliance with the STROBE criteria (Table 1).

**Table 1:** **Quality of clinical trials included in the present meta-analysis**

Concealment of allocation	Blinded investigator	Blinded participant	Blinded assessor	Blind data analysis	Intention-to-treat	Lost to follow-up
Adequate: 3	Yes: 3	Yes : 4	Yes : 3	Yes : 0	Declared : 3	Yes : 2
Inadequate: 1	No : 1	No: 1	No: 1	No: 2	Not declared: 2	No: 0
Obscure: 1	Obscure: 1	Obscure: 0	Obscure: 1	Obscure: 3		Obscure: 3

### Data extraction and statistical analysis

The studies were initially stratified according to their design. Based on these results, they were subsequently stratified following the Cochrane methodology. Review Manager 5 software, available from the Cochrane Collaboration, was used for statistical analysis. For dichotomous variables, the odds ratio (OR) with 95% confidence interval (CI) was calculated using random-effects and fixed-effects models. The Mantel–Haenszel chi-squared test and the I^2^ test were used to calculate heterogeneity [15].

## Results

**Figure 1:**
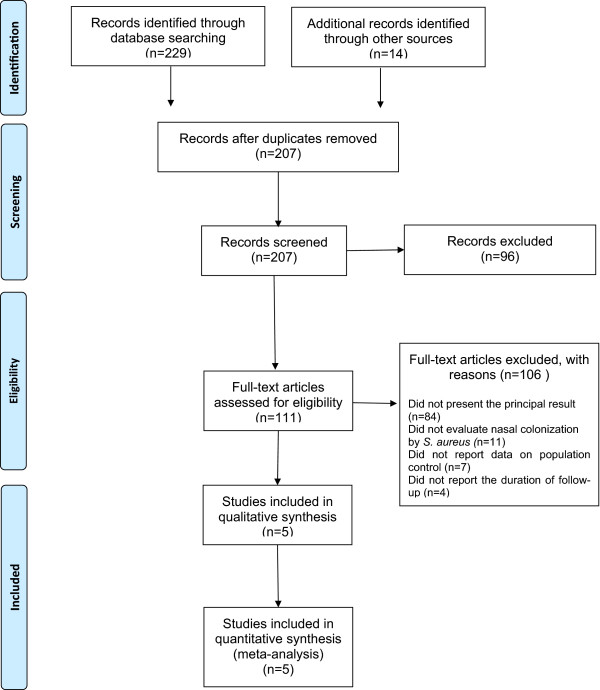
**PRISMA flow diagram of systematic review inclusion and exclusion process.**

PRISMA flow chart in Figure 1 summarises the search process. Initially, 143 articles were identified in the PubMed/Medline database, 54 in SciELO, 32 in Cochrane, 4 in LILACS, and 10 in Embase. Of the 243 total studies identified, 238 were excluded (36 were articles published and duplicated in different databases, 96 met the exclusion criteria, 84 did not present the principal result, 11 did not evaluate nasal colonization by *S. aureus*, 7 did not report data on population control, and 4 did not report the duration of follow-up).

Thus, five studies were evaluated: a prospective double-blind randomized controlled trial [16], three randomized clinical trials [17–19], and one historical cohort [20]. All studies were evaluated and classified as having a low risk of bias and adequate methodological quality by the Cochrane referential [13]. Randomization of the studies included in this review was performed by a computer, and concealment of the allocation was adequate.

These studies involved a total of 2,374 individuals (1,177 patients in the intervention group and 1,197 in the control group). Of these 2374 individuals, 626 (26.4%) were nasal carriers of *S. aureus* (see Additional file 1).

Based on the STROBE recommendations [13], three studies were placed in category A and two studies were placed in category B. This meta-analysis did not include studies in category C (<50% compliance with the criteria established by STROBE) (Table 1).

### *Screening of*S. aureus *in nasal carriers*

One study [20] provided strong evidence of a link between nasal colonization and bloodstream infection caused by *S. aureus*. In that study, patients who developed infection were transiently recolonized by *S. aureus* strains identical to the pretreatment colonized strains. The strains were confirmed to be identical by molecular typing and plasma DNA analysis.

Tracking methods that differed in frequency, location, and quantity of samples among the studies were used to identify *S. aureus* nasal carriers. One study [20] used three nasal samples (one before the intervention, one 3–5 days after the intervention, and one 10 days after the intervention; samples were reevaluated 1, 3, and 12 months after decolonization). Two studies [17, 18] used three to five nasal samples at the beginning and end of treatment. One study [16] measured IgG antibodies for *S. aureus* type 5 and 8 capsular polysaccharides for 2 years. Finally, one study [19] used nasal samples at the beginning of the study and upon suspicion of infection. Patients with three consecutive negative swabs were considered to be decolonized in most of the studies.

### Treatment/decolonization to control cross-transmission

Different interventions were evaluated, including topical antimicrobial agents applied at the catheter insertion site (2% mupirocin calcium ointment [17, 19] and 10% povidone-iodine solution [17, 18]) or in the nasal region (2% mupirocin calcium ointment [20]), bolus injection of cefotaxime through a central venous catheter [CVC] [19], and administration of an *S. aureus* vaccine containing type 5 and 8 capsular polysaccharides conjugated to nontoxic recombinant proteins [16] (see Additional file 1).

Mupirocin was compared with placebo with respect to eradication of colonization by *S. aureus* in three studies [17, 18, 20]. In total, 494 patients were evaluated (246 in the mupirocin group and 248 in the control group). Of all 494 patients, 147 (30%) were nasal carriers of *S. aureus*. The duration of treatment ranged from 140 to 570 days. Eradication of the nasal carrier state was successful in approximately 98% (range, 96–100%) of patients receiving mupirocin after 30 to 90 days of monitoring. One study [19] did not provide information about nasal colonization by *S. aureus* in the control group, and between-group comparison was therefore not possible. However, eradication of nasal colonization by *S. aureus* in the mupirocin group was 96% in that study.

**Figure 2:**
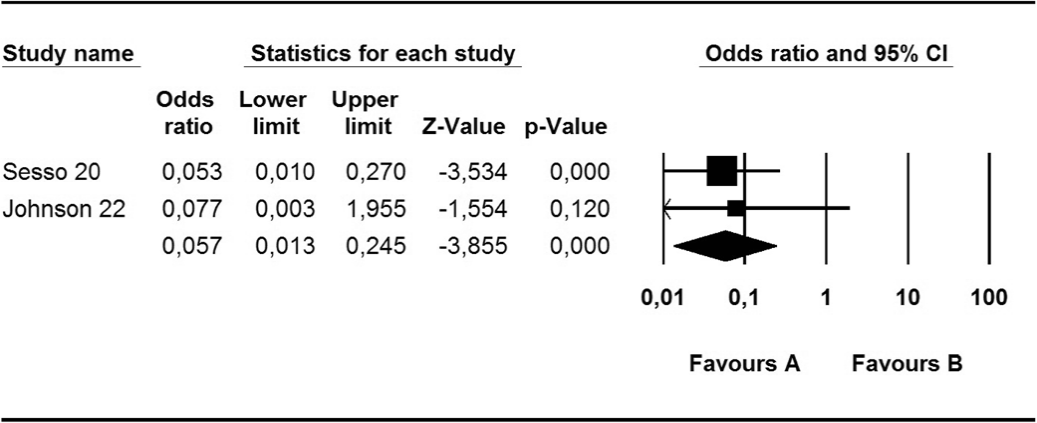
**Meta-analysis of mupirocin versus control: eradication of** ***S. aureus*** **nasal colonization in hemodialysis patients.**

The lack of heterogeneity in the results (Q, 0.01; I^2^, 0.00; p = 0.92) indicates that the probability of *S. aureus* nasal colonization in patients undergoing HD was 93% lower in mupirocin-treated patients than in untreated or placebo-treated patients [17, 18] (OR, 0.05; 95% CI, 0.01–0.27) (Figure 2).

Other treatments were encountered less frequently. Administration of a povidone-iodine solution eradicated nasal colonizationin 62% of carriers of *S. aureus* after 30 days of catheter placement [16], bolus catheter injection of cefotaxime eradicated methicillin-resistant *S. aureus* in 76% (n = 26/34) of patients [19], and administration of a conjugate vaccine containing *S. aureus* type 5 and 8 capsular polysaccharides offered protection against bacteremia by *S. aureus* in 90% of patients with antibody levels of ≥80 pg/mL (the estimated minimum level of protection) for up to approximately 40 weeks [16].

The acquisition of mupirocin resistance during treatment was reported in one study. Boelaert et al. [20] identified a strain of high-level mupirocin-resistant *S. aureus* (minimum inhibitory concentration of >512 yg/mL) after 19 months of mupirocin application among 29 strains isolated from patients colonized with *S. aureus*. The adverse events attributable to mupirocin use were mild and did not lead to discontinuation of treatment [17, 19, 20]. The incidence of local reactions such as malaise and myalgia was significantly higher in patients who received the vaccine than in patients in the control group, but they were mild or moderate and resolved within 2 days [16].

Table 2 provides details regarding author, year of publication, study population, total number of patients included, number of patients colonized with *S. aureus* (at entry into the study), number of patients with skin infection at the dialysis catheter insertion site, and bacteremia (see Additional file 1).

**Table 2:** **Tracking and management of patients with chronic kidney disease colonized with** ***S. aureus*** **undergoing hemodialysis through a central venous catheter**

Author	Year	Design	Patient (n)		NCSA (n/%)		Treatment		Follow up		Eradication	SIIS (n)		Bacteremia (n)	
			Treated group	Control group	Treated group	Control group	Treated group	Control group	Treated group	Control group		Treated group	Control group	Treated group	Control group
Hinefield^16^	2002	DBRCT	892	906	197 (22%)	200 (22%)	Vaccine	Placebo	17.5 months	17,2 months	86%	NT	NT	37	49
Sesso^20^	1998	RCT	69	67	28 (41%)	27 (40%)	Mupirocin CIS 3x/s	CIS	5 months		96%	5	24	2	11
								Povidine iodine							
Johnson^22^	2002	RCT	27	23	6 (22%)	6 (26%)	Mupirocin CIS 3x/w	Placebo	9 months		76%	0	5	1	5
Saxena^23^	2012	RCT	39	43	39 (100%)	43(100%)	Catheter bolus cefotaxime	Catheter bolus heparin	12 months		100%	7	9	12	22
Boelaert^30^	1993	HC	150	15	80 (53%)	0 (0%)	MUpirocin nasal3x\s	No treatment	18 months		96%	NT	NT	4	18

Note: n. number of patients; % Percentage; NCSA nasal carrier of *S. aureus*; SIIS: skin infection at the central venous catheter insertion site; DBRCT: Double blind randomized controlled trial; RCT: randomized clinical trial; HC: historical cohort; NT, not tested; CIS: catheter insertion site; 3x/w: three times per week.

### Prevention of skin infection at catheter insertion site and S. aureus bacteremia in patients undergoing HD

**Figure 3:**
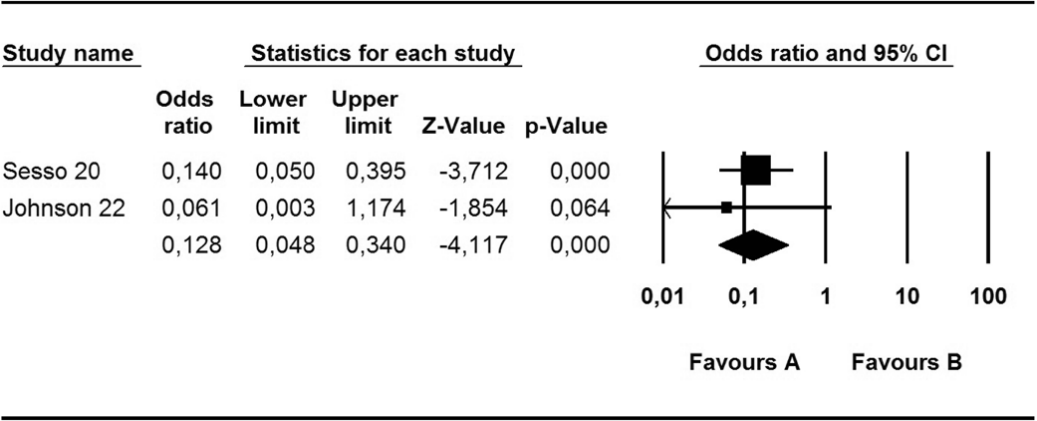
**Meta-analysis of mupirocin versus control:** ***S. aureus*** **skin infection at catheter insertion site.**

Figure 3 shows that the probability of *S. aureus* skin infection at the CVC insertion site for HD was 87% lower in the mupirocin group than in the control group (OR, 0.13; 95% CI, 0.05–0.34; p = 0.000). There was no significant heterogeneity among the studies (Q, 0.01; I^2^, 0.00; p = 0.92).

**Figure 4:**
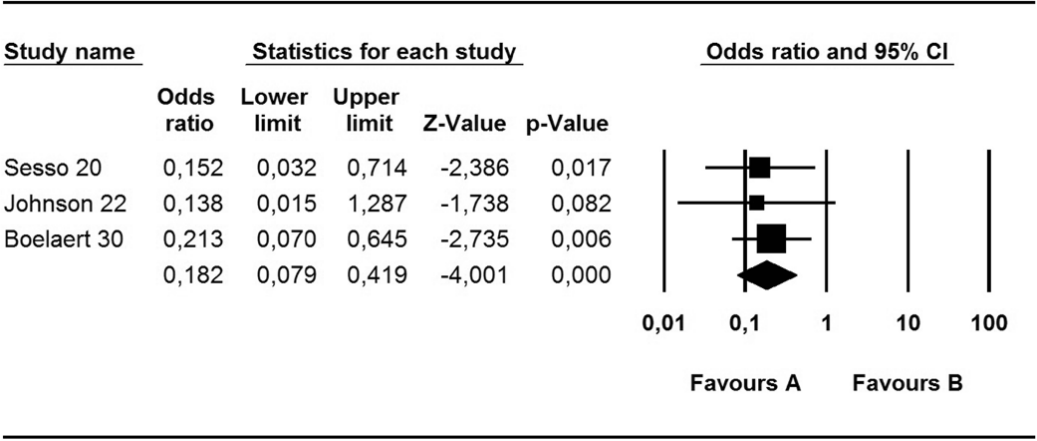
**Meta-analysis of mupirocin versus control: risk of** ***S. aureus*** **bacteremia.**

As shown in Figure 4, the risk of bacteremia in patients undergoing HD using a CVC was 82% lower in the mupirocin group than in the control group (OR, 0.18; 95% CI, 0.08–0.42. p < 0.0001). There was no significant heterogeneity among the studies (Q, 0.01; I^2^, 0.00; p = 0.92).

**Figure 5:**
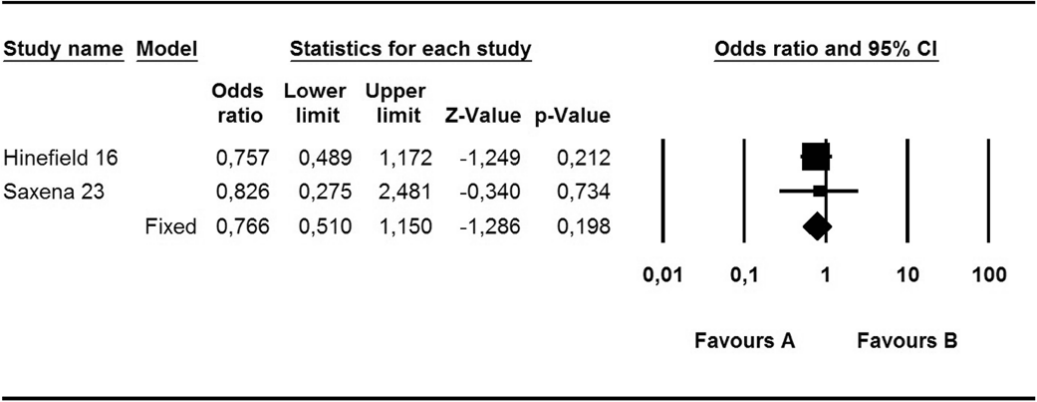
**Meta-analysis of diverse treatments versus control: risk of** ***S. aureus*** **bacteremia.**

Finally, no significant difference was observed in *S. aureus* bacteremia in patients undergoing HD with CVCs between the various treatments group (excluding mupirocin) and the control group (OR, 0.77; 95% CI, 0.51–1.15; p = 0.198) (Figure 5).

## Discussion

*Staphylococcus aureus* is the leading nosocomial pathogen worldwide [3]. Infection by *S. aureus* is associated with high morbidity and mortality rates [21]. Effective prevention strategies are essential because of the serious sequelae of this infection. Prevention of *S. aureus* infection has traditionally been focused on minimizing cross infection. However, it has been repeatedly shown that a high proportion of *S. aureus* infections originate from the nasal flora [3].

Nasal carriage of *S. aureus* is a known risk factor for subsequent infection in patients undergoing dialysis with intravascular devices [3]. The rates of *S. aureus* nasal colonization, skin infection at the exit of the CVC, and bacteremia were studied in the present review, which involved 2374 patients with CKD undergoing HD, 626 (26.4%) of whom were *S. aureus* nasal carriers.

Tracking methods for *S. aureus* nasal carrier identification differed in frequency, location, and quantity of samples among the studies in this meta-analysis. Only two studies [17, 18] used the same screening strategy; therefore, it is not possible to establish a strong, evidence-based, uniform recommendation regarding screening strategies.

In this review, mupirocin administration was the most effective treatment for eradication of the *S. aureus* nasal carrier state in the medium term (30–90 days); the estimated probability of success using mupirocin compared with no treatment was 93% (OR, 12.07; 95% CI, 0.01–0.40). The total estimated decolonization rate using other treatment methods was 76% from 4 to 40 weeks. The efficacy of mupirocin decreased with a prolonged follow-up period (>90 days). Moreover, a longer follow-up period after a short treatment period resulted in an increased risk of recolonization in other parts of the body [5]. Although *S. aureus* is found in other parts of the body beyond the nasal mucosa, its removal leads to loss of colonization in other locations such as the hands and skin; this indicates that the other body parts are infected via the nasal colonization [7].

Boelaert et al. [20] provided evidence for a link between nasal colonization and bloodstream infection caused by *S. aureus*. Patients who developed infections were transiently recolonized by *S. aureus* strains identical to the strains isolated before treatment; the strains were confirmed to be identical by molecular typing and analysis of DNA from plasma. The related infectious strains in the mupirocin group (n = 16 patients) differed from the original nasal strain. In contrast, four of the six infectious strains in the placebo group (n = 18 patients) were similar to the original nasal strain.

Despite improvements in HD techniques and CVC care, infectious complications remain the leading cause of death in patients undergoing HD [6]. Infection of the CVC site is primarily responsible for death in more than half of these patients [22].

A study involving 156 patients with CKD [6] demonstrated that the risk of bloodstream infection was 1.3 times higher in patients with positive skin cultures at the insertion site of the CVC for HD (relative risk, 1.29; 95% CI, 0.83–1.98; p < 0.001) and that the risk of bacteremia was 30% higher in patients with a positive culture at the site of HD catheter insertion (relative risk, 1.29; 95% CI, 0.83–1.98; p < 0.001).

In this review, mupirocin applied to the catheter exit site in *S. aureus* carriers was the most effective therapy and reduced the likelihood of skin infection at the catheter exit site by 87% in patients with CKD undergoing HD compared with no treatment or placebo (OR, 00.13; 95% CI, 0.05–0.34; p = 0.000). The risk of bacteremia in these patients was 82% lower in the mupirocin group than in the control group (OR, 00.18; 95% CI, 0.08–0.42; p < 0.0001).

Boelaert et al. [20] reported that the incidence of *S. aureus* bacteremia was four times lower after nasal decolonization with mupirocin in patients with end-stage renal disease than in the untreated group (0.024 vs. 0.097 per patient-year, p = 0.008). Johnson et al. [18] conducted a clinical trial involving patients undergoing HD and found a significantly lower incidence of bacteremia-related bloodstream infection (7% vs. 35%, p < 0.01) and longer survival duration (108 vs. 31 days, p < 0.05) in patients treated with dermal mupirocin at the CVC exit site three times a week than in untreated patients. Sesso et al. [17] performed a prospective randomized study and found that the proportion of patients with *S. aureus* skin infection at the CVC insertion site was lower in the mupirocin group than in the untreated group (4.3% vs. 23.9%, p = 0.001). In their study, *S. aureus* bacteremia was observed in 17 patients: 2 in the mupirocin group (0.71 episodes per 1,000 patient-days) and 15 in the control group (8.92 episodes per 1,000 patient-days; p < 0.001).

An issue of concern when mupirocin is used for long periods is the emergence of mupirocin resistance. However, resistance has not been reported when treatment is limited to mupirocin prophylaxis or healthy patients [3].

Three studies in the present meta-analysis evaluated resistance to mupirocin, and only one showed resistance associated with prolonged therapy. Notably, however, the long-term effect on the development of resistance is unknown because no studies have reported follow-up results of more than two Moreover, the techniques used varied among studies, and mupirocin resistance was not evaluated in most of them.

Recent studies of monitoring techniques, however, have reported that mupirocin-resistant *S. aureus* infections related to the use of CVCs for HD have led to a reduction in drug efficacy in the long term as well as emergence of resistance and recolonization by *S. aureus*. These are major causes of morbidity and mortality among these patients [10].

Alternatively, long-term studies have offered other treatment options to achieve *S. aureus* elimination, such as the proposed use of a conjugate vaccine that confers only partial immunity against *S. aureus* bacteremia for approximately 40 weeks in patients undergoing HD. After this time period, the protective effect decreases as the antibody levels began to decline [16].

One study showed that long-term cefotaxime blockade of CVCs in patients with concurrent end-stage renal disease and nasal colonization by *S. aureus* undergoing HD led to a significant overall reduction in the incidence of bacteremia (1.47 vs. 3.44 episodes per 1000 catheter-days, p < 0.001) and associated mortality (10.2% vs. 2.9%, p < 0.05) compared with heparin-only blockade of CVCs. This intervention is more effective against Gram-positive cocci (p = 0.032), including methicillin-sensitive *S. aureus* (p < 0.05). However, cefotaxime blockade provided no protection against methicillin-resistant *S. aureus* bacteremia and showed antimicrobial resistance associated with prolonged use [19].

There are some limitations in this meta-analysis. The type and duration of HD access were not provided in most studies. Thus, the differences in these potential risk factors for *S. aureus* catheter-site infection and bacteremia between cases and controls could not be evaluated. Additionally, the studies did not analyze *S. aureus* strains by typing or DNA analysis to confirm the association between nasal colonization and infection. Finally, no studies showed the results of a prolonged follow-up; therefore, it was not possible to investigate mupirocin resistance. The number of existing high-quality studies is too small to compare the effects of interventions other than the use of mupirocin. Local protocols and patient education interventions for the prevention of infection were not analyzed in the present study.

Notably, a major obstacle faced by these patients is delayed diagnosis using conventional microbiological culture methods. New screening methods such as real-time polymerase chain reaction allow for the detection of nasal colonization in <2 hours. Identification of nasal carriers is possible before infectious complications arise in this group of patients. Combining these screening methods with a short wait for effective treatment would allow for effective treatment of this group of high-risk patients.

## Conclusions

Colonization by *S. aureus* is common in patients undergoing chronic HD and occurred in 26.4% of such patients in this review. It is thus an important risk factor for the development of infections in these patients.

In this meta-analysis, regular clearance of *S. aureus* from the nose or use of a screening test to guide antibiotic therapy is recommended because the benefits of mupirocin ointment are relatively short-lived.

Mupirocin ointment effectively reduces the risk of infection at the HD venous catheter insertion site as well as the risk of catheter-related bacteremia. There are reports of resistance to mupirocin, and this should be considered in future studies with prolonged follow-up periods.

No other intervention has proven effective in the long term. Given the large number of patients undergoing HD and the importance of infectious complications in these patients, eradication of the carrier state of *S. aureus* seems justified. To date, topical mupirocin has exhibited the most proven beneficial effects. However, new alternatives should be further investigated in future randomized clinical trials to guide decisions regarding strategies for prevention of infections in patients undergoing chronic HD.

## Electronic supplementary material

Additional file 1: **PRISMA checklist.** (DOC 64 KB)
